# Gastric Adenocarcinoma Presenting as Chronic Back Pain: A Case Report

**DOI:** 10.5811/cpcem.2020.3.46660

**Published:** 2020-04-23

**Authors:** Alexandra Chitty, Dennis Cardriche, Thomas H. Matese

**Affiliations:** HCA Healthcare, St. Lucie Medical Center, Department of Emergency Medicine, Port St. Lucie, Florida

**Keywords:** Gastric adenocarcinoma, gastric cancer, back pain, chronic back pain

## Abstract

**Introduction:**

Early stage gastric cancer is usually asymptomatic. It is not until later stages of the disease, usually with metastasis, that patients typically develop symptoms that would prompt further evaluation.

**Case Report:**

We present a case of a patient with chronic back pain who was found to have a gastric antral mass as the etiology of her pain. The patient proceeded to have a partial gastrectomy with complete surgical excision of her early-stage gastric cancer, after which her chronic back pain resolved.

**Conclusion:**

This case demonstrates the importance of considering significant pathology in patients presenting with chronic complaints to the emergency department.

## INTRODUCTION

In 2016, back symptoms were the sixth most common reason for emergency department (ED) visits.^1^ Patients presenting with chronic back pain are often presumed to have a musculoskeletal etiology of pain. Multiple limb- or life-threatening diagnoses must be considered in these patients. Some of those diagnoses include acute spinal cord compression, cauda equina syndrome, aortic dissection, abdominal aortic aneurysm, and spinal infections, or hematomas. Another etiology commonly seen in the ED is back pain due to pathologic fractures of the spine secondary to metastatic disease. However, to our knowledge no case reports have been noted in the literature that describe early gastric cancer presenting with a primary symptom of back pain. We believe this is a unique case report.

## CASE REPORT

A 76-year-old female former smoker with a past medical history of hypertension and hypothyroidism presented to the ED at a community hospital with a chief complaint of intermittent left upper back pain for one year. The patient reported that she was not a local resident and had flown into the area four days prior. She reported acute worsening of her left upper back pain over the prior two days, localized the pain to underneath her left shoulder blade, and rated it as severe. She denied any obvious provoking factors and reported minimal relief when lying still on a heating pad. She had previously been seen by her primary care physician for the same complaint and had been started on celecoxib. The patient reported epigastric pain with celecoxib and noted that she had discontinued use.

The patient denied syncope, fever, chills, midline back pain, chest pain, dyspnea, cough, hemoptysis, nausea, vomiting, dysuria, hematochezia, and melena. She admitted to occasional heartburn. She was accompanied by her friend who reported that while out shopping the day prior, the patient had had a relatively brief episode where she became pale appearing and reported feeling weak, lightheaded, and nauseated. The patient had no surgical history and denied a history of alcohol use. She did report a 10-pound weight loss over the prior six months, which she attributed to grief over her husband’s recent death. She denied any specific trauma or injury but admitted to having lifted her husband multiple times prior to his death.

On arrival to the ED, the patient’s vital signs were reported as follows: temperature 36.9 degrees Celsius; heart rate 90 beats per minute; blood pressure 101/76 millimeters of mercury; respiratory rate 18 breaths per minute; pulse oximetry 100% on room air. On examination, the patient was alert, non-toxic appearing, and seemed to be in mild discomfort. Upon examination of the eyes, she was noted to have conjunctival pallor. Her lungs were clear to auscultation Although appearing mildly dyspneic, she demonstrated no other signs of respiratory distress. Her cardiovascular exam revealed a regular rate and rhythm with brisk capillary refill and equal pulses. Her abdomen was soft, non-distended with mild epigastric tenderness to palpation without guarding, rigidity, or evidence of peritoneal irritation. There was no palpable or pulsatile mass noted.

Her back appeared to be atraumatic without rash or skin discoloration. She had no midline vertebral tenderness to palpation and no tenderness was elicited upon palpation around the inferomedial aspect of the left scapula where the patient had reported the pain was located. There was no paraspinal tenderness or muscle spasm and no costovertebral tenderness. The patient had painless range of motion of her back. Her neurologic and extremity exams were unrevealing. A rectal exam demonstrated external hemorrhoids, normal sphincter tone, no saddle anesthesia, and no gross blood.

The patient’s vague symptoms, history, and the inability to duplicate the pain, coupled with the fact that the pain was severe and continual raised concern for a potentially serious etiology. We included pulmonary embolism, aortic dissection, aortic aneurysm, and acute coronary syndrome in the differential diagnosis that warranted further investigation.

A computed tomography angiography of the chest showed no evidence of pulmonary embolus, aortic aneurysm, aortic dissection, or any other acute pathology. A computed tomography of the abdomen and pelvis with intravenous (IV) contrast demonstrated stenosis of the celiac artery with post-stenotic dilation with no evidence of downstream ischemia. The only significant lab abnormality was a hemoglobin of 8.4 grams per deciliter (g/dL) (13.0–18.0 g/dL) and a hematocrit of 26.8% (40.0–52.0%) to which the patient denied a known history of anemia. A fecal occult blood test was noted to be positive.

A bleeding peptic ulcer was suspected as the etiology of the patient’s symptoms. However, given the acute worsening of pain two days prior, there was concern that a small peptic ulcer perforation could not be ruled out. She was treated with 40 milligrams of IV pantoprazole in the ED and was admitted to the hospital where she was evaluated by a gastroenterologist.

The following day, the patient’s hemoglobin dropped to 7.5 g/dL and her hematocrit dropped to 23%. The patient underwent an esophagogastroduodenoscopy and was found to have erosive gastritis with a large, ulcerated, and partially obstructing antral mass. The biopsy revealed infiltrating, poorly differentiated adenocarcinoma. The patient was subsequently evaluated by a general surgeon and underwent a robot-assisted distal gastrectomy (approximate 60% gastrectomy) with Billroth-II reconstruction. There was no evidence of metastatic disease intra-abdominally. The distal gastrectomy specimen that was removed measured 13 centimeters (cm) × 8 cm × 6 cm. The mucosal surface demonstrated a tan, fungating mass, measuring 6 cm × 4 cm. The final diagnosis was moderately differentiated (grade 2) infiltrating adenocarcinoma of intestinal type with tumor invasion into the muscularis propria. Thirty-six lymph nodes and all margins were free of tumor. The pathologic stage was pT2 pN0.

**CPC-EM Capsule**

What do we already know about this clinical entity?

Early symptoms of gastric cancer can be vague and non-specific.

What makes this presentation of disease reportable?

This is the first presentation reported in the literature of a patient with a primary complaint of back pain who was found to have early gastric cancer.

What is the major learning point?

This case highlights the importance of considering life-threatening diagnoses in patients who present with vague and even chronic complaints.

How might this improve emergency medicine practice?

This case demonstrates the importance of performing a thorough physical examination and having a high index of suspicion for serious pathology, especially in the elderly with vague complaints.

Following surgery, the patient underwent chemotherapy for three months and eventually stopped given the severity of side effects she had experienced. I spoke with her nine months following her ED visit and she reported that her chronic back pain did in fact resolve following surgical excision of her gastric cancer. The patient remains in complete remission.

## DISCUSSION

The American Cancer Society predicts that 27,600 cases of stomach cancer will be diagnosed in the United States in 2020 and 11,010 people will die from this type of cancer in the US in 2020.^2^ EDs often serve as the entrance into the healthcare system for those labeled with a first-time cancer diagnosis.^3^ Fifty-two percent of patients ultimately diagnosed with gastric cancer at one urban institution were admitted to the hospital from the ED.^3^ Those patients who were diagnosed after an ED visit were determined to have poorer survival estimates as these patients often had later stage disease.^3^ Because early symptoms of gastric cancer can be vague and non-specific, one should consider this diagnosis in patients presenting with poor appetite, weight loss, early satiety, nausea, anorexia, abdominal pain, dysphagia, or melena, as well as back pain or fatigue.^4^

Gastric cancer is not a diagnosis that is typically made in the ED, but ED evaluation can lead to admission for further evaluation of a potentially malignant process. Consider admitting patients who may be presenting with a first-time cancer diagnosis especially in those who lack reliable follow-up care, as early initiation of the diagnostic process and prompt therapeutic intervention can improve patient prognosis. It is prudent to keep such a diagnosis in mind and to broaden one’s differential diagnosis. While the focus of this case report was to highlight a unique presentation of early gastric cancer, we also emphasize the importance of a thorough physical examination, especially in those patients presenting with chronic complaints. For example, if a patient presenting with chronic back pain presumed to be of musculoskeletal nature does not have abnormalities on examination such as tenderness to palpation, significant muscle spasm, or pain on movement, consider that there may be some other pathology present.

A bleeding peptic ulcer with a possible microperforation was considered on ED evaluation of this patient, which prompted hospital admission. The patient did have multiple symptoms at presentation concerning for gastric cancer, many of them also consistent with a diagnosis of peptic ulcer disease. These symptoms included epigastric pain, occult gastrointestinal bleeding, unintentional weight loss, nausea, fatigue, and back pain. Fortunately, this patient was found to have early-stage gastric adenocarcinoma that was surgically resected in a timely fashion.

Upon review of the literature, we found a published case report that presents a patient with worsening back pain who was found to have lytic lesions in the vertebrae and who was ultimately found to have signet-ring cell gastric adenocarcinoma.^5^ However, we found no similar case reports of patients presenting primarily with back pain due to gastric cancer that were not associated with other pathologies, such as bony metastasis.

It seems that our patient’s back pain was likely a referred pain from the visceral autonomic nervous system. We already know that the nervous system plays a role in the development of cancer and that there is a complex relationship between pain and carcinogenesis.^6^ There are many algogenic mediators involved in the process of carcinogenesis that alter human pain pathways.^6^ This patient’s back pain resolved following excision of her gastric cancer.

## CONCLUSION

This case demonstrates the importance of gathering an appropriate history, performing a thorough physical examination, and considering non-musculoskeletal, serious pathology in elderly patients with a long pain history, vague complaints, and a physical examination that is contrary to a simple musculoskeletal problem. It is important to note that the function of the history and physical examination is to look for confirmatory and non-confirmatory elements that may help lead one to the correct diagnosis. Chronic back pain is a very common complaint in the ED. Taking a few extra minutes to listen to a patient and to perform a thorough examination can lead to a timely diagnosis of a life-threatening illness that can generate a better prognosis for the patient. We owe it to our patients to consider life-threatening illnesses, including cancer, during ED evaluation.
